# Scrofuloderma management with scar excision

**DOI:** 10.1016/j.jdin.2023.08.012

**Published:** 2023-08-26

**Authors:** McKenzie E. Maloney, Christina Lohmann, Brian Maloney

**Affiliations:** a Medical College of Georgia, Augusta University, Augusta, Georgia; b Department of Pathology, Northside Hospital, Atlanta, Georgia; c Department of Surgery, UGA/AU Medical Partnership, Atlanta, Georgia

**Keywords:** cutaneous tuberculosis, dermatologic surgery, scar excision, scar revision, scrofuloderma

## Challenge

Scrofuloderma is a rare extrapulmonary manifestation of tuberculosis, arising from contiguous spread from deeper structures.^1^ Subsequent scars are depressed, adherent, retractable, or keloidal.^2^ As these scars often occur in cosmetically sensitive areas, we propose surgical excision for esthetic improvement of scrofuloderma scaring.

## Solution

A 34-year-old woman from Morocco presented to the clinic to discuss scars from a scrofuloderma infection at the age of 4 years. At the time of active infection, several lesions in the neck area drained and erupted spontaneously, subsequently forming irregular, hyperpigmented, depressed scars. The lesions have remained asymptomatic since. She previously saw a provider in Morocco who did not feel comfortable excising the scars. Previous cholecystectomy scars healed very well.

**Fig 1:**
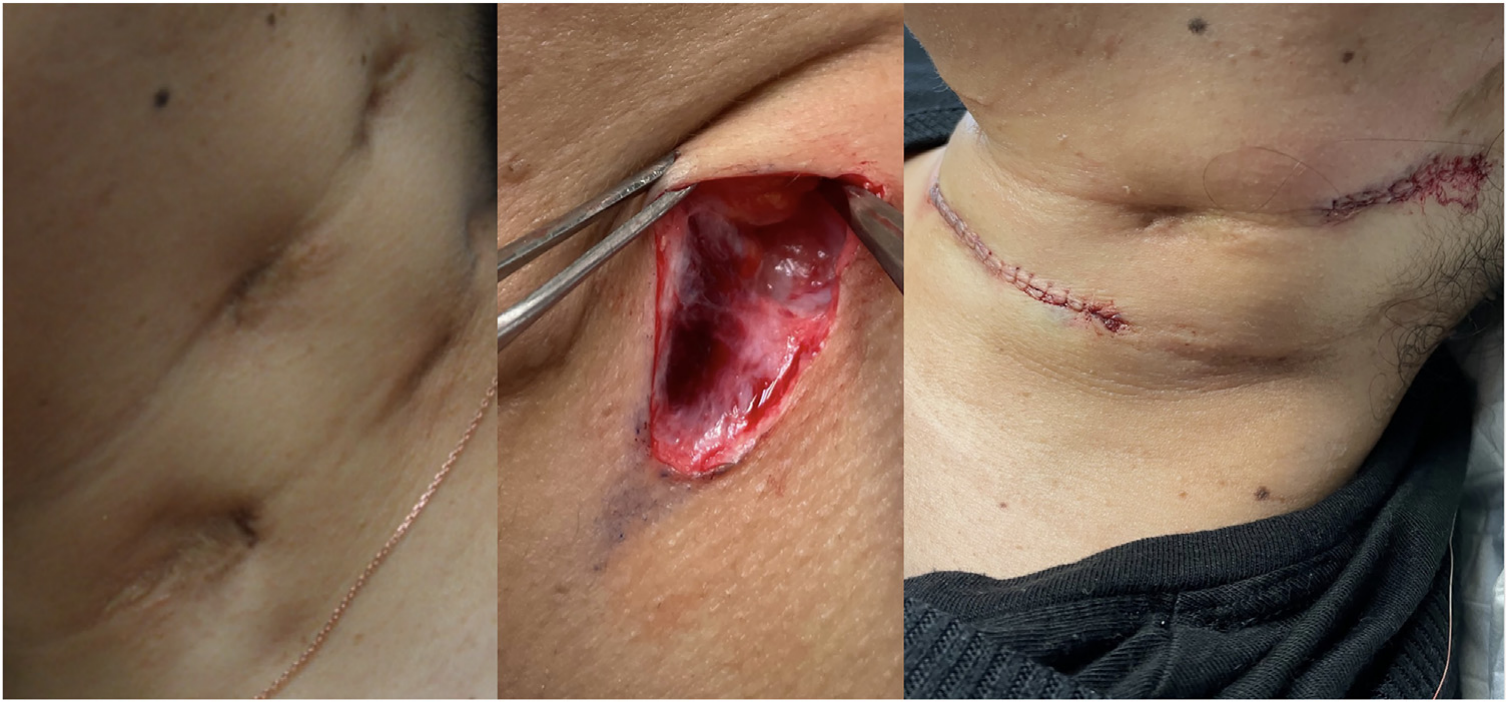
From left to right, scrofuloderma scars at presentation, deeper tracts seen during excision and immediate postoperative result.

On examination, 6 depressed, hyperpigmented, hypertrophic scars were present on the left side of the neck, measuring 2.2 cm by 1 cm (Fig 1). On the right, 4 similar scars were located over the cervical lymph nodes. On histology, epidermal papillomatosis with chronic inflammation around the hair follicles and focal areas of crateriform-like epidermal growth was seen. Biopsies were negative for acid-fast bacilli and fungi.

The patient was counseled on several methods of scar management including cutaneous bleaching, fillers, and fat transfer. The patient elected for scar excision in the office. Some of the scars were very close together and required staged excision.

After tissue infiltration with 1% lidocaine with epinephrine, the scars were excised. During the procedure, tracts extending into the deeper tissues were visualized (Fig 1). There was no external sinus or connection with the skin that would have suggested an epithelial lined tract. These tracts were not excised as it would have required significant dissection into the deeper neck structures. The scars were excised just below the level of the platysma muscle, leaving the deeper portions of the tract. The platysma was approximated with 5.0 vicryl sutures, and the skin was closed using a 5.0 fast-absorbing gut suture.

Postoperatively, the patient’s scars became hyperpigmented, and were managed with sun protection, topical 4% hydroquine twice a day, and systemic *Polypodium leucotomos* extract. Six weeks postoperatively, the scars displayed mild hypertrophy, managed with intralesional triamcinolone injections. The patient is very pleased with the results. Leaving the deeper tracts in place risks persistent depression following the excision. Other risks include infection, hyperpigmentation, and hypertrophic or keloidal scarring.

## Conflict of interest

None disclosed.
